# Transvaginal ovarian drilling prior to a second IVF cycle may improve the rate of euploidy in patients with polycystic ovarian syndrome when compared to controls

**DOI:** 10.1007/s10815-025-03778-x

**Published:** 2026-01-09

**Authors:** Moses Bibi, Sarah Rubin, Kaleb Noruzi, Rachel Stern, Shmuel Sashitzky, Adi Steinhart, Martin D. Keltz

**Affiliations:** 1 Department of Obstetrics and Gynecology, Westchester Medical Center, New York Medical College, 35 Sunshine Cottage Road, Valhalla, NY USA; 2 WestMed Reproductive Services, Purchase, NY USA

**Keywords:** PCOS, IVF, PGT-A, Infertility, TVOD, Euploid

## Abstract

**Purpose:**

To assess the impact of transvaginal ovarian drilling (TVOD) on euploidy rates following repeat in vitro fertilization (IVF) in subjects with polycystic ovarian syndrome (PCOS).

**Materials and methods:**

A single institution retrospective cohort study between January 2017 and December 2024, all patients with PCOS, as confirmed by Rotterdam criteria, who had TVOD performed prior to a repeat IVF cycle and underwent PGT-A in both the prior and repeat cycles were included and compared to a well-matched control. The primary outcome was the number of transferable embryos per cycle. Secondary outcomes included: blastocyst yield, euploid yield, and rates of aneuploidy.

**Results:**

Eighteen subjects met criteria in our study time frame and were compared to 57 control subjects. The mean age for the TVOD and control groups was 35.3 ± 4.6 and 36.5 ± 4.4 respectively (*p* = 0.34). TVOD was associated with a doubling of blastocyst yield, a sixfold increase in the yield of euploid blasts, and a nearly fourfold decrease in the percentage of aneuploid blasts. When compared to controls, TVOD resulted in a significant improvement in the yield of euploid embryos, from + 1.3 to + 2.4 (*p* = 0.01), and transferable embryos from + 1.5 to + 3.9 (*p* = 0.001). TVOD also resulted in a decrease in the percentage of aneuploid embryos from − 8.5 to − 49% (*p* < 0.001).

**Conclusion:**

TVOD appears to have positively impacted the yield of transferable embryos and euploid embryos in patients with PCOS when compared to a well-matched control.

**Supplementary Information:**

The online version contains supplementary material available at 10.1007/s10815-025-03778-x.

## Introduction

Polycystic ovary syndrome (PCOS) is the most prevalent endocrine disorder affecting 10–15% of the reproductive-aged female population. It is characterized by polycystic appearing ovaries on ultrasound, hyperandrogenism, and menstrual irregularities [1]. It is frequently associated with metabolic conditions such as diabetes and obesity and is a leading cause of anovulatory infertility [2]. Up to 80% of women with PCOS experience infertility primarily due to ovulatory dysfunction [1].

The treatment of infertility in PCOS is initiated with ovulation induction using oral agents such as letrozole or clomiphene citrate (CC) [3, 4]. When insulin resistance or poor response to low-dose oral agents is encountered, metformin may be added, as it has been shown to improve ovulation rates with letrozole or CC [5]. Although CC successfully induces ovulation in 50–90% of cases, 15–20% of patients remain anovulatory even at high doses. These CC-resistant patients require alternative treatments to achieve ovulation and pregnancy [6]. In vitro fertilization (IVF) is indicated for primary treatment in severe male factor and tubal factor and is often employed for patients who do not respond to or fail to conceive with oral ovulation induction [7]. Despite its efficacy, IVF in PCOS patients is associated with complications such as ovarian hyperstimulation syndrome (OHSS) and increased rates of aneuploidy, lowering its age-adjusted efficacy [8–12]. Additionally, pregnancy in PCOS patients is associated with increased rates of spontaneous abortion, gestational diabetes, and adverse obstetric outcomes [2].

Preimplantation genetic testing for aneuploidy (PGT-A) has been utilized as a screening tool to identify euploid and aneuploid embryos in patients undergoing IVF [13]. The introduction of next-generation sequencing (NGS) to PGT-A has increased the sensitivity and resolution of detecting copy number variations by chromosome, thereby improving embryo selection and reducing the time to singleton live birth [14].

Laparoscopic ovarian diathermy (LOD), or electrosurgical drilling, was initially developed as a minimally invasive alternative to laparotomy with drilling or wedge resection to induce ovulation in CC-resistant PCOS patients. While effective, LOD's invasiveness and risk of pelvic adhesions limit its popularity [15–17]. Transvaginal ovarian drilling (TVOD), an incision-free alternative, was reported in 2001 to improve IVF outcomes and reduce hyperstimulation in CC-resistant PCOS patients. TVOD has shown profound effects on androgen levels and ovulatory function, equivalent to LOD, but with fewer complications [7, 18]. In 2010, our group presented a pilot study of TVOD for select CC-resistant patients with recurrent IVF failure [19, 20]. In 2008, we began a prospective time-series cohort study presenting our initial findings that TVOD markedly improved blastulation and ongoing pregnancy rates in CC-resistant PCOS patients with a history of IVF failure [21]. We hypothesized that these improvements may be attributed to an increased rate and yield of euploid or transferable mosaic embryos.

This study aims to evaluate the impact of TVOD on the percent and yield of transferable embryos in PCOS patients undergoing IVF and PGT-A when compared to our control. We hypothesize that TVOD will significantly enhance the proportion of both euploid and transferable mosaic blastocysts as it markedly improves the blastulation rate [20]. As we have previously shown, euploidy and a lesser degree of aneuploidy are associated with a higher blastulation rate [22].

## Materials and methods

We conducted a single-institution retrospective cohort study. Approval from the Institutional Review Board (IRB) was obtained prior to the initiation of the study. The Electronic Medical Record (EMR) at our institution was screened between January 2017 and December 2024 for all patients who met the inclusion criteria.

Inclusion criteria for all patients in this study were women aged 18 to 45 years, with a diagnosis of PCOS confirmed by the Rotterdam criteria, as defined by at least two out of three of the following criteria: (1) polycystic-appearing ovaries with at least 12 peripheral follicles seen on sagittal or transverse ovarian ultrasound image; (2) androgen excess as defined as any hyperandrogenic signs or symptoms, such as acne, hirsutism, or hyperandrogenemia; and (3) menstrual irregularities resulting in fewer than 8 menses per year. All subjects underwent an initial IVF cycle at our institution within 3 months prior to TVOD. Each case subject underwent a subsequent IVF cycle immediately following TVOD. Both IVF cycles included PGT-A using NGS. All subjects were on metformin at the time of TVOD. All case subjects had two or fewer euploid embryos in their pre-TVOD PGT-A IVF cycle. TVOD was offered to all patients who met criteria for PCOS, who had failed implantation following embryo transfer with only one or fewer euploid embryos remaining, and who were planning to undergo a repeat cycle.

Patients were then compared to a control group that included all patients in the same time period, between 2017 and 2024, aged 18 to 45 years old, who met Rotterdam criteria for PCOS and had an AMH greater than or equal to 3.5 ng/mL. Every patient in the control group underwent an initial and subsequent freeze-all PGT-A IVF cycle within 12 months of each other in this time frame, wherein the initial cycle 2 or fewer euploid embryos were yielded. Patients included in the control had received no TVOD; however, they had failed implantation following embryo transfer with only one of fewer euploid embryos. Three patients in the control group received omnitrope during their IVF cycle; however, when comparing those who received omnitrope to those who did not, there was no statistical difference in IVF outcomes. Therefore, all patients, including those who received omnitrope, were included in the control group for comparison.

In the menstrual cycle prior to IVF, patients received oral contraceptive pills (OCPs) in an OCP–gonadotropin-releasing hormone agonist overlap protocol. After at least 7 days of OCPs, patients began 10 units of subcutaneous leuprolide acetate along with the OCP for 4 days. OCPs were then discontinued, and withdrawal bleeding occurred. In the pre-drilling cycles, gonadotropin stimulation commenced following the completion of menses. In the post-drilling cycles, ovarian drilling was performed after the completion of menstrual flow and 1–3 days prior to the commencement of controlled ovarian hyperstimulation (COH) for IVF.

TVOD was performed in an ambulatory surgical center under Propofol anesthesia. Patients were placed in the dorsal lithotomy position, and the vagina was prepped with a sterile Povidone-iodine solution. Transvaginal ultrasound was used to assess the position of the ovaries. A 17-gauge, 35-cm retrieval needle with constant suction at 170 mm Hg was used for drilling. Each ovary was punctured once and penetrated to its full thickness 100 times, moving from the lateral to the medial surface of each ovary with visualization of the iliac vein and artery throughout the procedure. Patients were observed for 1 h following anesthesia and then discharged home. No significant bleeds were noted following the procedure.

For both the pre- and post-drilling cycles, COH was initiated with exogenous gonadotropins and patients received 10 units of subcutaneous leuprolide acetate daily until hCG administration. The dose of gonadotropins, recombinant FSH (rFSH), and/or human menopausal gonadotropins (HMG), was individually adjusted based on ovarian response. Transvaginal sonography and serial estradiol (E2) levels were used to monitor ovarian follicular development. Once a dominant follicle reaches a diameter of 18 to 20 mm, 2500–10,000 IU of hCG is administered based on the patient’s individual weight and risk for OHSS. Transvaginal ultrasound-guided oocyte retrieval was performed 36 h later. Insemination was performed with either standard insemination or intracytoplasmic sperm injection (ICSI) based on male factor indications. Fertilization was confirmed 20 h later by the presence of two pronuclei (2PN).

Embryos were kept in culture until days 3–4, when they underwent laser-assisted hatching. Day 5–7 blastocysts underwent trophectoderm biopsy and were subsequently frozen. Biopsy samples were sent to CooperGenomics (CooperSurgical Fertility Solutions, Livingston, NJ) for PGT-A testing, which reported back NGS data.

The primary outcome was the number of transferable embryos per cycle, defined as the sum of euploid and safe-to-transfer mosaic embryos. Previous studies, including our own data, have shown the transfer of mosaic embryos has non-inferior live birth outcomes when compared to euploid embryos [23]. Secondary outcomes included total blastocyst yield, euploid embryo yield, percent blastulation, and rates of euploidy, mosaicism, and aneuploidy. Additional data evaluated include age, body mass index (BMI), race, gravidity, and parity. Statistical significance in our primary and secondary outcomes was defined as *p* ≤ 0.01 due to the small sample size and multiple significance testing. Trends were reported at *p* < 0.05. Continuous data were compared using the paired *t*-test and Wilcoxon signed rank test for patients’ own pre- and post-drilling cycles. Categorical data were compared using Fisher’s exact test. To compare the IVF outcomes for the TVOD and control group, the delta was calculated as the difference between the second and first IVF cycle for the treatment group and the controls; the treatment group versus control deltas underwent statistical comparison utilizing *t*-test or Wilcoxon rank sum test.

Regression modeling was performed to assess for potential confounding effects of baseline characteristics on the association of TVOD with the delta of IVF outcomes, or change in outcomes from the first to second IVF cycle.Normally distributed outcomes were modeled with linear regression. Regression coefficients (*β*), 95% confidence intervals (CIs), and *p*-values were reported for each predictor, and *F*-statistics, *F*-statistic *p*-values, *R*^2^ values, and adjusted *R*^2^ values were reported for each model. Non-normally distributed outcomes were modeled with Gamma regression if right-skewed or Inverse Gaussian regression if left-skewed. For skewed outcomes that included negative or zero values, a constant was added to shift the distribution to positive, as these models can only handle positive values. Rate ratios, 95% CIs, and *p*-values were reported for each predictor, while Chi-square statistics and *p*-values of likelihood ratio tests (LRT) to assess model fit were reported for each model. First, univariate analysis was performed to evaluate the isolated association between TVOD and outcomes. Multivariate analysis was subsequently performed to assess the impact of age, anti-mullerian hormone (AMH) level, BMI, male factor infertility, and change in total gonadotropin dosage from the first to second IVF cycle on the association between TVOD and outcomes. Race and ethnicity were not included in this analysis because the proportion of unknown race and ethnicity was highly unbalanced between groups, limiting meaningful adjustment. However, a sensitivity analysis including only patients with known race and ethnicity was performed, in which race and ethnicity were included as a covariate with the aforementioned baseline characteristic variables. White race/ethnicity was used as the reference category. All statistics were performed using R version 4.3.2.

## Results

Eighteen subjects met the inclusion criteria as TVOD cases, while 57 patients met the criteria for our controls. Our TVOD group had a mean age of 35.3 ± 4.6 years as compared to our control group with a mean age of 36.5 ± 4.4 (*p* = 0.34), as shown in Table 1. The TVOD and control groups had a mean BMI of 29.7 ± 6.0 and 27.2 ± 6.8, respectively (*p* = 0.07). There was no difference in mean Anti-Müllerian Hormone (AMH) level between the TVOD group and the control group with 5.6 ± 2.6 ng/mL as compared to 5.1 ± 2.1 ng/mL (*p* = 0.48). The distributions of race and ethnicity in the TVOD and control groups were significantly different.There was a higher proportion of Hispanic patients in our TVOD group (33.3%) as compared to our control (7.0%). There was also a larger proportion of Asian patients in our TVOD group (28%) as compared to our control (5.2%). There was a lower proportion of patients that identified as White in our TVOD group. Notably, 32% of our control patients had an unknown race and ethnicity. In the TVOD group, 83.3% of patients were nulliparous as compared to 68.4% in the control group (*p* = 0.37). In the TVOD group, 44.4% of patients were nulligravid as compared to 51% in the control group (*p* = 0.79). Furthermore, 88.9% (16/18) of patients in the TVOD group had ICSI performed as compared to 87.7% (50/57) in the control group (*p* = 0.79). In the TVOD group, 44.4% (8/18) of patients were affected by male factor infertility as compared to 43.9% (25/57) (*p* = 0.6). There was no difference in insemination method, intracytoplasmic sperm injection (ICSI) or conventional insemination (CI), between groups in both the first and second cycle. There was also no difference in insemination method between the first and second cycles within the TVOD group (*p* = 0.66) and control group (*p* = 0.44). The TVOD group received a significantly higher total gonadotropin dosage than the control group in both the first and second cycles.

**Table 1:** Patient baseline characteristics

Parameter	TVOD group (*N* = 18) Mean (SD) [min, max]/*n* (%)	Control group (*N* = 57) Mean (SD) [min, max]/*n* (%)	*p*-value
Age	35.3 (4.7) [27, 42]	36.5 (4.4) [26, 45]	0.34
Race & Ethnicity			0.005*
Asian	5/18 (28%)	3/57 (5.2%)	
Black	1/18 (5.6%)	2/57 (3.5%)	
Hispanic	6/18 (33.3%)	4/57 (7.0%)	
White	6/18 (33.3%)	30/57 (52.6%)	
Unknown	0/18 (0%)	18/57 (32%)	
BMI	29.7 (6.0) [21, 39.5]	27.2 (6.8) [18.5, 42]	0.07
Gravida			0.79
Nulligravid	8 (44.4%)	29 (50.9%)	
Gravid	10 (55.6%)	28 (49.1%)	
Parity			0.37
Nulliparous	15 (83.3%)	39 (68.4%)	
Parous	3 (16.7%)	18 (31.6%)	
AMH level (ng/mL)	5.6 (2.6) [2.7, 12.2]	5.1 (2.1) [3.51, 13.4]	0.48
Male Factor	8 (44.4%)	25 (43.9%)	1.0
First cycle insemination method			0.75
ICSI	14 (77.8%)	46 (80.7%)	
CI	4 (22.2%)	11 (19.3%)	
Second cycle insemination method			1.0
ICSI	16 (88.9%)	50 (87.7%)	
CI	2 (11.1%)	7 (12.3%)	
First cycle total gonadotropin dosage (IU)	4241.2 (1331.3) [950–7575]	3318.7 (1030.0) [1849.5–5100]	0.009*
Second cycle total gonadotropin dosage (IU)	4365.8 (1279.0) [1700–6500]	3483.8 (1221.6) [1386–5725]	0.01*
Delta of total gonadotropin dosage between cycles (IU)	124.59 (1193.89)	165.14 (886.98)	0.89

*statistically significant

However, there was no significant difference in total gonadotropin dosage between the first and second cycles within the TVOD group (0.66) and control group (*p* = 0.61). There was also no significant difference in the change in total gonadotropin dosage between cycles.

There was no difference in the number of oocytes retrieved prior to and following TVOD at 21.7 ± 8.2 and 21.8 ± 6.8 (*p* = 0.94). The number of 2PN embryos following TVOD increased from 10.9 ± 5.2 to 14.2 ± 6.0 (*p* = 0.004) as shown in Table 2. Both the number of and percentage of blastocysts increased following TVOD as shown in both Tables 2 and 3.

**Table 2:** Paired outcomes for IVF cycles immediately before and after TVOD as compared to the control (embryo yield)

Outcome measure	TVOD group			Control group		
	Pre-TVOD mean (SD)	Post-TVOD mean (SD)	Confidence interval, *p*-value	First cycle mean (SD)	Second cycle mean (SD)	Confidence interval, *p*-value
2PN yield	10.9 (5.2)	14.2 (6.0)	[− 5.5, − 1.2] 0.004*	8.6 (4.3)	10.5 (5)	[− 3.3, − 0.5] 0.008*
Blastocysts yield	3.0 (2.1)	6.9 (4.8)	[− 6.0, − 1.5] 0.002*	2.4 (1.9)	4.2 (3.2)	[− 2.7, − 1] < 0.001*
Euploid yield	0.56 (0.7)	2.94 (1.9)	[− 3.5, − 2] < 0.001*	0.33 (0.48)	1.6 (1.7)	[− 1.75, − 0.84] < 0.001*
Transferable embryo yield	0.72 (0.8)	4.7 (3.1)	[− 5.5, − 2.5] < 0.001*	0.72 (0.9)	2.2 (2)	[− 2.1, − 0.97] < 0.001*
Aneuploid yield	2.2 (2.0)	1.2 (1.6)	[− 0.5, 2.5] 0.1	1.54 (1.5)	2.0 (2.1)	[− 0.96, 0.02] 0.05

*statistically significant

**Table 3:** Paired outcomes for IVF cycles immediately before and after TVOD as compared to the control (percentage of embryos)

Outcome measure	TVOD group			Control group		
	Pre-TVOD mean (SD)	Post-TVOD mean (SD)	Confidence interval, *p*-value	First cycle mean (SD)	Second cycle mean (SD)	Confidence interval, *p*-value
Percent Blastocysts	28.9% (18)	49.3% (20.5)	[− 34%, − 7%] 0.004*	30% (23)	39% (24)	[− 12%, − 1%] 0.03
Percent Euploid	20% (33)	47.9% (30)	[− 60%, − 10%] 0.01*	12% (22)	31% (28)	[− 27%, − 11%] < 0.001*
Percent Transferable	24% (33)	73% (28.3)	[− 80%, − 30%] 0.002*	27% (35)	46% (36)	[− 29%, − 8%] < 0.001*
Percent Aneuploid	67.3% (37)	18.5% (27)	[33%, 80%] 0.002*	54.9% (41)	46.3% (34)	[− 3%, 20%] 0.14

*statistically significant

TVOD was also associated with a significant increase in the number and percentage of euploid blastocysts. The yield of euploid blastocysts increased sixfold, from 0.56 ± 0.7 to 2.9 ± 1.9 (*p* < 0.001). The percent of euploid blastocysts increased more than twofold, from 20 ± 33% to 48 ± 30% (*p* = 0.01). Furthermore, the number of mosaic blastocysts increased following TVOD from 0.16 ± 0.4 to 1.7 ± 2.1 (*p* = 0.007). The percentage of transferable embryos, the combined percentage of euploid and mosaic blastocysts, increased over threefold from 24 ± 33% to 73 ± 28% (*p* = 0.002). Similarly, the combined number of euploid and mosaic blastocysts increased significantly following TVOD from 0.72 ± 0.8 to 4.7 ± 3.1 (*p* < 0.001). The percentage of aneuploids created decreased significantly, from 67.3 ± 37% to 18.5 ± 27% (*p* = 0.002). However, there was a decreased trend in the number of aneuploid embryos following TVOD from 2.2 ± 2.0 to 1.2 ± 1.6 (*p* = 0.1).

When analyzing IVF outcomes for our control group comparing their first and second IVF cycles in a 12-month period, there was an improved trend in the number of oocytes retrieved, 15.3 ± 6 to 17.3 ± 6.5 (*p* = 0.02) and an increase in the number of 2PN per cycle from 8.6 ± 4.3 to 10.5 ± 5 (*p* = 0.008) in the subsequent cycle as shown in Table 2. In our control, there was also improvement in the number and percentage of euploid blastocysts, 0.33 ± 0.48 to 1.6 ± 1.7 (*p* < 0.001) and 12.4 ± 22% to 31.3 ± 28% (*p* < 0.001) as shown in Tables 2 and 3. There was also an improvement in the yield and percentage of transferable embryos, 0.72 ± 0.9 to 2.2 ± 2 (*p* = 0.001) and 27 ± 35% to 46 ± 36% (*p* < 0.001). There was no effect on aneuploid embryo yield or percentage among cycles in the control group.

When comparing TVOD cases to the control group using an absolute comparison, the difference between the second and first cycle, there was an increase in the number of transferable embryos of + 3.9 ± 3.2, which was a significantly greater increase than the significant increase in controls of + 1.5 ± 2.1 (*p* < 0.001), as shown in Table 4. There was also an increase in the percentage of transferable embryos per blastocyst from + 18 ± 39% in controls to + 49 ± 45% with TVOD (*p* = 0.008). Similarly, there was an increase in the number of euploid embryos from + 1.3 ± 1.7 in controls to + 2.4 ± 1.6 with TVOD (*p* = 0.01). There were no changes in the 2PN yield, blastocyst yield, percent blastocysts, or euploidy rate with the addition of TVOD. Furthermore, there was a highly significant decrease in both the number and percentage of aneuploid embryos with TVOD as compared to the controls, + 0.47 ± 1.8 to − 1.0 ± 2.8 (*p* = 0.01) in aneuploid yield and − 8.5 ± 43% to − 48.9 ± 43% (*p* < 0.001) in percent aneuploidy, as shown in Table 4.

**Table 4:** Delta comparison: change from the first to the second IVF cycle (TVOD group compared to controls)

Outcome measure	Second cycle change in TVOD Mean (SD)	Second cycle change in control Mean (SD)	*p*-value
2PN yield	+ 3.3 (4.3)	+ 1.9 (5.2)	0.39
Blastocyst yield	+ 3.9 (5.3)	+ 1.9 (3.2)	0.11
Percent blastocysts	+ 20% (26)	+ 9.4% (31)	0.18
Euploid yield	+ 2.4 (1.6)	+ 1.3 (1.7)	0.01*
Percent euploid	+ 28.0% (41)	+ 18% (39)	0.32
Transferable embryo yield	+ 3.9 (3.2)	+ 1.5 (2.1)	< 0.001*
Percent transferable	+ 49% (45)	+ 18% (39)	0.008*
Aneuploid yield	− 1.0 (2.8)	+ 0.47 (1.8)	0.01*
Percent aneuploid	− 48.9% (43)	− 8.5% (43)	< 0.001*

*statistically significant

Univariate regression analysis revealed a significant association between TVOD and a greater increase in euploid and transferable embryo yield and transferable embryo rate from first to second IVF cycle when compared to controls. This analysis also revealed a significant association between TVOD and a greater reduction in aneuploid embryo yield and rate between cycles when compared to controls. After adjusting for age, AMH, BMI, male factor infertility, and change in total gonadotropin dosage from first to second cycle with multivariate regression, the association between TVOD and the relative change in the yield and rate of transferable and aneuploid embryos remained significant. Compared to controls, TVOD was associated with a 46% greater increase in transferable embryo yield and a 16% greater increase in transferable embryo rate between cycles. TVOD was also associated with a 19% greater reduction in aneuploid embryo yield between cycles when compared to controls, as well as a 43 percentage-point greater reduction in aneuploid embryo rate. The association between TVOD and change in euploid embryo yield trended towards a greater increase from first to second cycle when compared to controls, but did not reach statistical significance as shown in Table 5.

**Table 5:** Multivariate analysis: effect of TVOD on the change in outcomes from the first to the second IVF cycle

Outcome measure	Second cycle change in TVOD Mean (SD)	Second cycle change in control Mean (SD)	Effect (*β*-coefficient/rate ratio) [CI]	*p*-value	Adjusted effect^a^ (*β*-coefficient/rate ratio) [CI]	Adjusted *p*-value
Oocyte yield			RR = 0.90 [0.76, 1.08]	0.25	RR = 0.88 [0.73, 1.06]	0.17
2PN yield	+ 3.3 (4.3)	+ 1.9 (5.2)	RR = 1.10 [0.92, 1.34]	0.32	RR = 1.07 [0.89, 1.30]	0.47
Blastocyst yield	+ 3.9 (5.3)	+ 1.9 (3.2)	*β* = 0.37 [− 0.0061, 0.75]	0.054	*β* = 0.28 [− 0.10, 0.67]	0.15
Percent blastocysts	+ 20% (26)	+ 9.4% (31)	*β* = 0.11 [− 0.051, 0.27]	0.18	*β* = 0.078 [− 0.087, 0.24]	0.35
Euploid yield	+ 2.4 (1.6)	+ 1.3 (1.7)	RR = 1.33 [1.03, 1.74]	0.035*	RR = 1.26 [0.97, 1.67]	0.097
Percent euploid	+ 28.0% (41)	+ 18% (39)	*β* = 0.09 [− 0.089, 0.27]	0.32	*β* = 0.098 [− 0.087, 0.28]	0.30
Transferable embryo yield	+ 3.9 (3.2)	+ 1.5 (2.1)	RR = 1.53 [1.21, 1.96]	< 0.001*	RR = 1.46 [1.15, 1.87]	0.0029*
Percent transferable	+ 49% (45)	+ 18% (39)	RR = 1.16 [1.04, 1.32]	0.013*	RR = 1.16 [1.03, 1.32]	0.018*
Aneuploid yield	− 1.0(2.8)	+ 0.47 (1.8)	RR = 0.84 [0.75, 0.96]	0.011*	RR = 0.81 [0.71, 0.92]	0.0022*
Percent aneuploid	− 48.9% (43)	− 8.5% (43)	*β* = − 0.40 [− 0.63, − 0.17]	< 0.001*	*β* = − 0.43 [− 0.66, − 0.20]	< 0.001*

*statistically significant

^a^Adjusted for age, AMH, BMI, male factor infertility, change in total gonadotropin dosage from the first to the second IVF cycle

A separate multivariate regression analysis including only patients with known race and ethnicity (excluding 18 of 57 control patients) with race and ethnicity added as a covariate revealed that TVOD remained significantly associated with relative changes in transferable embryo rate and aneuploid embryo yield and rate from the first to second IVF cycles compared to controls. TVOD was associated with a 34 percentage-point greater increase in transferable embryo rate from first to second cycle compared to controls (*β* = 0.34 [0.036, 0.63], *p* = 0.029). TVOD was associated with a 23% greater reduction in aneuploid embryo yield (RR = 0.77 [0.65, 0.93], *p* = 0.0087) and a 50 percentage-point greater reduction in aneuploid embryo rate (*β* = − 0.50 [− 0.77, − 0.22], *p* < 0.001) between cycles when compared to controls. TVOD remained associated with a greater increase in transferable embryo yield from the first to second cycle when compared to controls (RR = 1.30 [0.96, 1.79], *p* = 0.091) but was no longer statistically significant, likely due to decreased power because of the smaller sample size included in the sensitivity analysis. (Supplemental Tables 1–10).

## Discussion

In this retrospective cohort study of patients with PCOS, transvaginal ovarian drilling (TVOD) resulted in a sixfold increase in the yield of euploid blastocysts and a twofold increase in the rate of euploid blastocysts relative to an initial failed IVF PGT-A cycle yielding fewer than two euploid embryos. Similarly, there was a 6.5-fold increase in transferable embryo yield. A control group yielding fewer than two euploid embryos in an initial cycle also had significantly improved IVF outcomes in a subsequent cycle within a year. While both the TVOD and control group had an increase in the embryo and transferable embryo yield, TVOD resulted in a significantly greater increase in transferable embryos compared to the control group, even after adjustment for possible confounders. Furthermore, TVOD was associated with a significantly greater reduction in the aneuploid embryo percentage and yield compared to the control group. These findings support the use of TVOD for PCOS patients who are resistant to CC with a poor outcome after an initial IVF cycle, as it appears to both increase transferable embryo yield and decrease aneuploidy rates per blastocyst.

Previous studies have reported that PCOS is associated with poor IVF outcomes [22]. Patients with PCOS undergoing IVF have been shown to have increased rates of aneuploidy and mosaicism in embryos in addition to lower fertilization rates [2, 10, 24]. Following IVF, patients with PCOS have also been shown to have significantly increased risks of miscarriage, biochemical pregnancy loss, and OHSS [10]. TVOD has been shown to improve ovulatory function and androgen levels, which are critical factors in the development of high-quality oocytes and embryos [7, 15]. Our results align closely with our group’s prior findings in a prospective cohort study, which also demonstrated improved blastulation rates, implantation rates, and ongoing pregnancy rates in CC-resistant PCOS patients following TVOD [20]. This consistency across studies reinforces the potential benefits of TVOD in improving reproductive outcomes in PCOS patients with prior IVF.

TVOD is a preferred alternative to historical laparoscopic ovarian drilling techniques, such as laparoscopic ovarian diathermy (LOD) or electrosurgical drilling. While LOD has shown to be effective, it is invasive with an increased risk of pelvic adhesions and cautery/potential damage to the ovary [15–17]. Alternatively, transvaginal ovarian drilling (TVOD), an incision-free alternative, has shown profound effects on androgen levels and ovulatory function, equivalent to LOD, but with fewer complications [7, 18].

The mechanisms by which TVOD improves egg and embryo quality in PCOS patients are not fully understood, but several hypotheses have been suggested in the literature. By reducing androgen levels in the ovarian environment, TVOD may improve the health of the follicle and the granulosa cell-oocyte complex, leading to the development of higher-quality oocytes and embryos [18, 25, 26]. We propose that TVOD may enhance ovarian blood flow and oxygenation, which are essential for follicular development and oocyte maturation. We have an ongoing prospective evaluation to assess this hypothesis utilizing Doppler ultrasound with total ovarian blood flow and vascular resistance measurements before and after TVOD; early data from our group suggests improved blood flow to the ovary, which may be the link to improved euploidy and diminished aneuploidy rates as reported in this study. We recommend further studies assessing the measurements of PO2 and androgen levels in follicular fluid pre and post-TVOD to further elucidate the physiologic mechanism by which TVOD improves IVF outcomes. One additional consideration is if stimulating the stroma of the polycystic ovary via TVOD is beneficial; it is possible that oocyte retrieval, although to a lesser degree, may exert a beneficial effect, which may explain why even the control group had improved outcomes, in combination with regression to the mean.

Our study has several limitations. The most significant limitation is the small sample size, which may limit the external validity of our findings. However, subjects represented a range of age groups and ethnic and racial backgrounds. To generate confidence that these results are applicable to a broader PCOS population prior to an initial IVF, a multicenter trial with a larger sample size is necessary. Enrollment at a single institution is slow, as there are few patients who are appropriate candidates for TVOD. A further limitation is the retrospective nature of the study, which can increase the risk of bias as to who may have received TVOD at the time as opposed to patients who did not receive the intervention. One example of this is differences in first cycle performance between our TVOD and control group. Additionally, it is possible that changes in the ovulation induction regimens played a role in improved outcomes as well; therefore, not all benefits may be derived from TVOD alone. Another consideration is that there are cycle-to-cycle variations in ploidy; however, attempts to mitigate this were through comparison to our control. Though, due to the retrospective nature of the study, it is possible that some of the benefit from TVOD may be derived from regression to the mean as well as other unmeasured confounders not accounted for in the multivariate regression analysis. Of note, 32% of the control group had unknown race/ethnicity, which did not allow for complete analysis of the potential confounding effect of race and ethnicity on the association of TVOD and IVF outcomes. However, a sensitivity analysis including only patients with known race and ethnicity was carried out, which produced results largely consistent with the main multivariate analysis despite decreased statistical power. When comparing the TVOD outcomes to the control group, it is possible that 38% of the change in the TVOD group may have been attributable to regression to the mean. Our research is ongoing in PCOS and as we significantly increase our sample size, we will continue to report our findings.

Despite these limitations, our study has notable strengths. Conducting the study at a single institution ensures consistency in the procedures performed and makes it easier to track and monitor data. Furthermore, being at a single center, we have included every patient at our practice who has received TVOD in this study, lowering selection bias. Additionally, the outcomes of our current study are consistent with our prior reported outcomes on TVOD from a different institution and patient base, which speaks to the consistency and generalizability of our data [20]. Additionally, our data showed that post-TVOD cycles among an initially poor prognosis group resulted in an average of an additional 2.9 euploid embryos per retrieval. This yield far exceeds the mean of 1.88 euploid embryos found in good prognosis patients undergoing their first IVF cycle in prior studies [27].

Transvaginal ovarian drilling appeared to improve euploid and transferable embryo yield and decrease the number of aneuploid blastocysts produced. We suspect these findings are likely a product of improved oxygenation of the ovary during the stimulation process. Future prospective studies with larger sample sizes and randomization are warranted to further validate these findings and to explore the long-term clinical benefits of incorporating TVOD in the initial IVF treatment protocols for PCOS patients.

## Conclusion

TVOD appears to have positively impacted both euploid and transferable embryo yield and decreased aneuploid blastocyst yield in patients with PCOS when compared to a control. TVOD, as applied in the context of patients with PCOS who have failed to conceive in IVF, may be considered an adjuvant to improve outcomes in subsequent IVF attempts by increasing transferable embryo yield; however, further larger and prospective studies are necessary to better understand the extent of this effect.

## Supplementary Information

Below is the link to the electronic supplementary material.

ESM 1 (DOCX 25.6 KB)

ESM 2 (MOV 59.0 MB)

## Data Availability

The original contributions presented in this study are included in the article. Further inquiries can be directed to the corresponding author.
