# Supplementary material for: Transvaginal ovarian drilling prior to a second IVF cycle may improve the rate of euploidy in patients with polycystic ovarian syndrome when compared to controls
Source: J Assist Reprod Genet. 2026 Jan 9;43(3):779–87. doi: 10.1007/s10815-025-03778-x (PMC12982796; doi:10.1007/s10815-025-03778-x)
Supplement: Supplementary file 1 — (DOCX 25.6 KB) [file 10815_2025_3778_MOESM1_ESM.docx]

Supplemental Table 1 - Regression analysis of the association of TVOD with the change in total oocytes retrieved from first to second IVF cycle

| Model Type | Variable | **Effect** (β-coefficient/Rate Ratio)  [CI] | P-value | F-statistic/Chi-square (X^2^) Statistic,  R^2^,  Adjusted R^2^ | F-statistic/LRT P-Value |
| --- | --- | --- | --- | --- | --- |
| Univariate, Gamma | TVOD | RR=0.90 [0.76,1.08] | 0.25 | X^2^=0.15 | 0.25 |
| Multivariate,  Gamma | TVOD | RR=0.88 [0.73,1.06] | 0.17 | X^2^=0.59 | 0.53 |
|  | Age | RR=1.00 [0.98,1.01] | 0.67 |  |  |
|  | AMH | RR=1.02 [0.99,1.06] | 0.18 |  |  |
|  | BMI | RR=1.00 [0.99,1.02] | 0.54 |  |  |
|  | Male Factor | RR=0.95 [0.82,1.11] | 0.55 |  |  |
|  | Change in Total Gonadotropin Dosage Between Cycles | RR=0.99 [0.99,1.00] | 0.27 |  |  |
| Multivariate with Race/  Ethnicity,  Gamma | TVOD | RR=0.86 [0.67,1.11] | 0.24 | X^2^=0.78 | 0.73 |
|  | Age | RR=1.00 [0.98,1.02] | 0.85 |  |  |
|  | AMH | RR=1.03 [0.98,1.08] | 0.25 |  |  |
|  | BMI | RR=1.01 [0.99,1.02] | 0.50 |  |  |
|  | Male Factor | RR=0.97 [0.79,1.19] | 0.79 |  |  |
|  | Change in Total Gonadotropin Dosage Between Cycles | RR=0.99 [0.99,1.00] | 0.41 |  |  |
|  | Asian Race/Ethnicity | RR=0.89 [0.65,1.25] | 0.49 |  |  |
|  | Black or African American Race/Ethnicity | RR=1.14 [0.72,1.87] | 0.59 |  |  |
|  | Hispanic/Latino  Race/Ethnicity | RR=0.99 [0.73,1.36] | 0.95 |  |  |

Supplemental Table 2 - Regression analysis of the association of TVOD with the change in 2PN yield from first to second IVF cycle

| Model Type | Variable | **Effect** (β-coefficient/Rate Ratio)  [CI] | P-value | F-statistic/Chi-square (X^2^) Statistic,  R^2^,  Adjusted R^2^ | F-statistic/LRT P-Value |
| --- | --- | --- | --- | --- | --- |
| Univariate, Gamma | TVOD | RR=1.10 [0.92,1.34] | 0.32 | X^2^=0.13 | 0.31 |
| Multivariate,  Gamma | TVOD | RR=1.07 [0.89,1.30] | 0.47 | X^2^=0.93 | 0.28 |
|  | Age | RR=1.00 [0.98,1.02] | 0.99 |  |  |
|  | AMH | RR=1.03 [1.00,1.08] | 0.050 |  |  |
|  | BMI | RR=1.01 [0.99,1.02] | 0.36 |  |  |
|  | Male Factor | RR=0.90 [0.77,1.06] | 0.21 |  |  |
|  | Change in Total Gonadotropin Dosage Between Cycles | RR=0.99 [0.99,1.00] | 0.77 |  |  |
| Multivariate with Race/  Ethnicity,  Gamma | TVOD | RR=1.12 [0.89,1.42] | 0.32 | X^2^=1.53 | 0.14 |
|  | Age | RR=1.01 [0.98,1.03] | 0.57 |  |  |
|  | AMH | RR=1.04 [1.00,1.09] | 0.078 |  |  |
|  | BMI | RR=1.01 [0.99,1.02] | 0.21 |  |  |
|  | Male Factor | RR=0.94 [0.77,1.14] | 0.51 |  |  |
|  | Change in Total Gonadotropin Dosage Between Cycles | RR=1.00 [0.99,1.00] | 0.53 |  |  |
|  | Asian Race/Ethnicity | RR=0.84 [0.63,1.15] | 0.27 |  |  |
|  | Black or African American Race/Ethnicity | RR=1.19 [0.77,1.89] | 0.45 |  |  |
|  | Hispanic/Latino  Race/Ethnicity | RR=0.77 [0.57,1.04] | 0.093 |  |  |

Supplemental Table 3 - Regression analysis of the association of TVOD with the change in blastocyst yield from first to second IVF cycle

| Model Type | Variable | **Effect** (β-coefficient/Rate Ratio)  [CI] | P-value | F-statistic/Chi-square (X^2^) Statistic,  R^2^,  Adjusted R^2^ | F-statistic/LRT P-Value |
| --- | --- | --- | --- | --- | --- |
| Univariate, Linear | TVOD | β=0.37 [-0.0061,0.75] | 0.054 | F=3.85  R^2^=0.050  Adjusted R^2^=0.037 | 0.054 |
| Multivariate,  Linear | TVOD | β=0.28 [-0.10,0.67] | 0.15 | F=1.54  R^2^=0.12  Adjusted R^2^=0.042 | 0.18 |
|  | Age | β=-0.013 [-0.050,0.023] | 0.47 |  |  |
|  | AMH | β=0.060 [-0.015,0.13] | 0.11 |  |  |
|  | BMI | β=0.017 [-0.0079,0.042] | 0.18 |  |  |
|  | Male Factor | β=-0.11 [-0.44,0.21] | 0.50 |  |  |
|  | Change in Total Gonadotropin Dosage Between Cycles | β=-1.05x10^-5^  [-0.00015,0.00013] | 0.88 |  |  |
| Multivariate with Race/  Ethnicity,  Linear | TVOD | β=0.16 [-0.35,0.68] | 0.53 | F=0.85  R^2^=0.14  Adjusted R^2^=-0.025 | 0.57 |
|  | Age | β=-0.027 [-0.076,0.022] | 0.27 |  |  |
|  | AMH | β=0.042 [-0.059,0.14] | 0.41 |  |  |
|  | BMI | β=0.011 [-0.022,0.045] | 0.50 |  |  |
|  | Male Factor | β=-0.11 [-0.55,0.34] | 0.63 |  |  |
|  | Change in Total Gonadotropin Dosage Between Cycles | β=0.00010  [-0.00015,0.00025] | 0.62 |  |  |
|  | Asian Race/Ethnicity | β=0.22 [-0.46,0.91] | 0.51 |  |  |
|  | Black or African American Race/Ethnicity | β=0.79 [-0.23,1.82] | 0.13 |  |  |
|  | Hispanic/Latino  Race/Ethnicity | β=0.083 [-0.60,0.76] | 0.81 |  |  |

Supplemental Table 4 - Regression analysis of the association of TVOD with the change in blastocyst rate from first to second IVF cycle

| Model Type | Variable | **Effect** (β-coefficient/Rate Ratio)  [CI] | P-value | F-statistic/Chi-square (X^2^) Statistic,  R^2^,  Adjusted R^2^ | F-statistic/LRT P-Value |
| --- | --- | --- | --- | --- | --- |
| Univariate, Linear | TVOD | β=0.11 [-0.051,0.27] | 0.18 | F=1.86  R^2^=0.025  Adjusted R^2^=0.011 | 0.18 |
| Multivariate,  Linear | TVOD | β=0.078 [-0.087,0.24] | 0.35 | F=1.21  R^2^=0.097  Adjusted R^2^=0.017 | 0.31 |
|  | Age | β=-0.011 [-0.026,0.0051] | 0.18 |  |  |
|  | AMH | β=0.0017 [-0.030,0.034] | 0.92 |  |  |
|  | BMI | β=0.0075 [-0.0032,0.018] | 0.16 |  |  |
|  | Male Factor | β=0.0060 [-0.13,0.15] | 0.93 |  |  |
|  | Change in Total Gonadotropin Dosage Between Cycles | β=2.74x10^-5^  [-3.45x10^-5^,8.93x10^-5^] | 0.38 |  |  |
| Multivariate with Race/  Ethnicity,  Linear | TVOD | β=0.0087 [-0.20,0.22] | 0.93 | F=0.67  R^2^=0.11  Adjusted R^2^=-0.055 | 0.73 |
|  | Age | β=-0.017 [-0.037,0.0030] | 0.094 |  |  |
|  | AMH | β=-0.0049 [-0.046,0.036] | 0.81 |  |  |
|  | BMI | β=0.0066 [-0.0070,0.020] | 0.33 |  |  |
|  | Male Factor | β=-0.019 [-0.20,0.16] | 0.84 |  |  |
|  | Change in Total Gonadotropin Dosage Between Cycles | β=-2.61x10^-6^  [-8.48x10^-5^ ,7.95x10^-5^] | 0.95 |  |  |
|  | Asian Race/Ethnicity | β=0.15 [-0.13,0.42] | 0.29 |  |  |
|  | Black or African American Race/Ethnicity | β=0.079 [-0.34,0.49] | 0.70 |  |  |
|  | Hispanic/Latino  Race/Ethnicity | β=0.11 [-0.17,0.38] | 0.43 |  |  |

Supplemental Table 5 - Regression analysis of the association of TVOD with the change in euploid yield from first to second IVF cycle

| Model Type | Variable | **Effect** (β-coefficient/Rate Ratio)  [CI] | P-value | F-statistic/Chi-square (X^2^) Statistic,  R^2^,  Adjusted R^2^ | F-statistic/LRT P-Value |
| --- | --- | --- | --- | --- | --- |
| Univariate, Gamma | TVOD | RR=1.33 [1.03,1.74] | 0.035* | X^2^=1.17 | 0.028 |
| Multivariate,  Gamma | TVOD | RR=1.26 [0.97,1.67] | 0.097 | X^2^=2.45 | 0.13 |
|  | Age | RR=0.99 [0.96,1.02] | 0.48 |  |  |
|  | AMH | RR=1.03 [0.98,1.09] | 0.21 |  |  |
|  | BMI | RR=1.01 [0.99,1.03] | 0.13 |  |  |
|  | Male Factor | RR=1.02 [0.81,1.28] | 0.88 |  |  |
|  | Change in Total Gonadotropin Dosage Between Cycles | RR=0.99 [0.99,1.00] | 0.45 |  |  |
| Multivariate with Race/  Ethnicity,  Gamma | TVOD | RR=1.13 [0.79,1.63] | 0.48 | X^2^=1.85 | 0.65 |
|  | Age | RR=0.99 [0.95,1.02] | 0.46 |  |  |
|  | AMH | RR=1.03 [0.97,1.11] | 0.37 |  |  |
|  | BMI | RR=1.01 [0.99,1.04] | 0.23 |  |  |
|  | Male Factor | RR=0.94 [0.69,1.28] | 0.67 |  |  |
|  | Change in Total Gonadotropin Dosage Between Cycles | RR=0.99 [0.99,1.00] | 0.34 |  |  |
|  | Asian Race/Ethnicity | RR=1.19 [0.78,1.88] | 0.46 |  |  |
|  | Black or African American Race/Ethnicity | RR=0.84 [0.43,1.76] | 0.62 |  |  |
|  | Hispanic/Latino  Race/Ethnicity | RR=1.17 [0.73,1.89] | 0.51 |  |  |

Supplemental Table 6 - Regression analysis of the association of TVOD with the change in euploid rate from first to second IVF cycle

| Model Type | Variable | **Effect** (β-coefficient/Rate Ratio)  [CI] | P-value | F-statistic/Chi-square (X^2^) Statistic,  R^2^,  Adjusted R^2^ | F-statistic/LRT P-Value |
| --- | --- | --- | --- | --- | --- |
| Univariate, Linear | TVOD | β=0.09 [-0.089,0.27] | 0.32 | F=1.01  R^2^=0.014  Adjusted R^2^=0.00019 | 0.32 |
| Multivariate,  Linear | TVOD | β=0.098 [-0.087,0.28] | 0.30 | F=0.91  R^2^=0.014  Adjusted R^2^=0.00019 | 0.50 |
|  | Age | β=-0.0031 [-0.021,0.015] | 0.73 |  |  |
|  | AMH | β=-0.011 [-0.047,0.025] | 0.55 |  |  |
|  | BMI | β=-0.00070 [-0.013,0.011] | 0.16 |  |  |
|  | Male Factor | β=-0.0062 [-0.16,0.15] | 0.94 |  |  |
|  | Change in Total Gonadotropin Dosage Between Cycles | β=-6.98x10^-5^  [-0.00014,-2.95x10^-7^] | 0.050 |  |  |
| Multivariate with Race/  Ethnicity,  Linear | TVOD | β=0.12 [-0.11,0.35] | 0.29 | F=1.57  R^2^=0.23  Adjusted R^2^=0.084 | 0.15 |
|  | Age | β=0.00066 [-0.021,0.023] | 0.95 |  |  |
|  | AMH | β=-0.032 [-0.077,0.013] | 0.16 |  |  |
|  | BMI | β=-0.0014 [-0.016,0.014] | 0.85 |  |  |
|  | Male Factor | β=-0.082 [-0.28,0.12] | 0.41 |  |  |
|  | Change in Total Gonadotropin Dosage Between Cycles | β=-0.00012  [-0.00021 ,-2.77x10^-5^] | 0.012 |  |  |
|  | Asian Race/Ethnicity | β=0.16 [-0.14,0.46] | 0.30 |  |  |
|  | Black or African American Race/Ethnicity | β=-0.39 [-0.85,0.064] | 0.090 |  |  |
|  | Hispanic/Latino  Race/Ethnicity | β=-0.14 [-0.44,0.16] | 0.35 |  |  |

Supplemental Table 7 - Regression analysis of the association of TVOD with the change in transferable embryo yield from first to second IVF cycle

| Model Type | Variable | **Effect** (β-coefficient/Rate Ratio)  [CI] | P-value | F-statistic/Chi-square (X^2^) Statistic,  R^2^,  Adjusted R^2^ | F-statistic/LRT P-Value |
| --- | --- | --- | --- | --- | --- |
| Univariate, Gamma | TVOD | RR=1.53 [1.21,1.96] | <0.001 | X^2^=2.69 | <0.001 |
| Multivariate,  Gamma | TVOD | RR=1.46 [1.15,1.87] | 0.0029 | X^2^=3.99 | 0.0027 |
|  | Age | RR=1.00 [0.97,1.02] | 0.68 |  |  |
|  | AMH | RR=1.06 [1.01,1.11] | 0.021 |  |  |
|  | BMI | RR=1.00 [0.99,1.02] | 0.72 |  |  |
|  | Male Factor | RR=0.99 [0.81,1.22] | 0.95 |  |  |
|  | Change in Total Gonadotropin Dosage Between Cycles | RR=1.00 [0.99,1.00] | 0.85 |  |  |
| Multivariate with Race/  Ethnicity,  Gamma | TVOD | RR=1.30 [0.96,1.79] | 0.091 | X^2^=3.18 | 0.074 |
|  | Age | RR=1.00 [0.97,1.03] | 0.91 |  |  |
|  | AMH | RR=1.05 [0.99,1.12] | 0.083 |  |  |
|  | BMI | RR=1.00 [0.99,1.00] | 0.94 |  |  |
|  | Male Factor | RR=0.91 [0.70,1.20] | 0.50 |  |  |
|  | Change in Total Gonadotropin Dosage Between Cycles | RR=1.00 [0.99,1.00] | 0.43 |  |  |
|  | Asian Race/Ethnicity | RR=1.25 [0.85,1.88] | 0.28 |  |  |
|  | Black or African American Race/Ethnicity | RR=1.34 [0.75,2.54] | 0.35 |  |  |
|  | Hispanic/Latino  Race/Ethnicity | RR=1.11 [0.74,1.67] | 0.62 |  |  |

Supplemental Table 8 - Regression analysis of the association of TVOD with the change in transferable embryo rate from first to second IVF cycle

| Model Type | Variable | **Effect** (β-coefficient/Rate Ratio)  [CI] | P-value | F-statistic/Chi-square (X^2^) Statistic,  R^2^,  Adjusted R^2^ | F-statistic/LRT P-Value |
| --- | --- | --- | --- | --- | --- |
| Univariate, Inverse Gaussian | TVOD | RR=1.16 [1.04,1.32] | 0.013 | X^2^=0.17 | 0.0081 |
| Multivariate,  Inverse Gaussian | TVOD | RR=1.16 [1.03,1.32] | 0.018 | X^2^=0.21 | 0.22 |
|  | Age | RR=0.99 [0.98,1.01] | 0.46 |  |  |
|  | AMH | RR=1.01 [0.98,1.03] | 0.54 |  |  |
|  | BMI | RR=1.00 [0.99,1.00] | 0.33 |  |  |
|  | Male Factor | RR=0.99 [0.90,1.10] | 0.95 |  |  |
|  | Change in Total Gonadotropin Dosage Between Cycles | RR=1.00 [0.99,1.00] | 0.87 |  |  |
| Multivariate with Race/  Ethnicity,  Linear | TVOD | β=0.34 [0.036,0.63] | 0.029 | F=1.18  R^2^=0.18  Adjusted R^2^=0.028 | 0.33 |
|  | Age | β=0.0068 [-0.021,0.035] | 0.63 |  |  |
|  | AMH | β=0.0035 [-0.055,0.062] | 0.90 |  |  |
|  | BMI | β=-0.011 [-0.031,0.0081] | 0.25 |  |  |
|  | Male Factor | β=-0.15 [-0.40,0.11] | 0.26 |  |  |
|  | Change in Total Gonadotropin Dosage Between Cycles | β=-2.59x10^-5^  [-9.16x10^-5^,-0.00014] | 0.66 |  |  |
|  | Asian Race/Ethnicity | β=0.12 [-0.27,0.52] | 0.54 |  |  |
|  | Black or African American Race/Ethnicity | β=-0.056 [-0.65,0.54] | 0.85 |  |  |
|  | Hispanic/Latino  Race/Ethnicity | β=-0.070 [-0.46,0.32] | 0.72 |  |  |

Supplemental Table 9 - Regression analysis of the association of TVOD with the change in aneuploid yield from first to second IVF cycle

| Model Type | Variable | **Effect** (β-coefficient/Rate Ratio)  [CI] | P-value | F-statistic/Chi-square (X^2^) Statistic,  R^2^,  Adjusted R^2^ | F-statistic/LRT P-Value |
| --- | --- | --- | --- | --- | --- |
| Univariate, Gamma | TVOD | RR=0.84 [0.75,0.96] | 0.011 | X^2^=0.38 | 0.010 |
| Multivariate,  Gamma | TVOD | RR=0.81 [0.71,0.92] | 0.0022 | X^2^=1.02 | 0.0085 |
|  | Age | RR=0.99 [0.98,1.00] | 0.095 |  |  |
|  | AMH | RR=0.98 [0.96,1.01] | 0.18 |  |  |
|  | BMI | RR=1.00 [0.99,1.02] | 0.052 |  |  |
|  | Male Factor | RR=0.92 [0.82,1.03] | 0.15 |  |  |
|  | Change in Total Gonadotropin Dosage Between Cycles | RR=1.00 [0.99,1.00] | 0.80 |  |  |
| Multivariate with Race/  Ethnicity,  Inverse Gaussian | TVOD | RR=0.77 [0.65,0.93] | 0.0087 | X^2^=0.17 | 0.029 |
|  | Age | RR=0.98 [0.96,1.00] | 0.024 |  |  |
|  | AMH | RR=0.96 [0.93,1.00] | 0.034 |  |  |
|  | BMI | RR=1.01 [0.99,1.03] | 0.055 |  |  |
|  | Male Factor | RR=0.93 [0.80,1.10] | 0.41 |  |  |
|  | Change in Total Gonadotropin Dosage Between Cycles | RR=0.99 [0.99,1.00] | 0.78 |  |  |
|  | Asian Race/Ethnicity | RR=0.98 [0.79,1.23] | 0.86 |  |  |
|  | Black or African American Race/Ethnicity | RR=0.95 [0.67,1.44] | 0.80 |  |  |
|  | Hispanic/Latino  Race/Ethnicity | RR=0.98 [0.78,1.25] | 0.89 |  |  |

Supplemental Table 10 - Regression analysis of the association of TVOD with the change in aneuploid rate from first to second IVF cycle

| Model Type | Variable | **Effect** (β-coefficient/Rate Ratio)  [CI] | P-value | F-statistic/Chi-square (X^2^) Statistic,  R^2^,  Adjusted R^2^ | F-statistic/LRT P-Value |
| --- | --- | --- | --- | --- | --- |
| Univariate, Linear | TVOD | β=-0.40 [-0.63,-0.17] | <0.001 | F=12.02  R^2^=0.14  Adjusted R^2^=0.13 | <0.001 |
| Multivariate,  Linear | TVOD | β=-0.43 [-0.66,-0.20] | <0.001 | F=3.44  R^2^=0.23  Adjusted R^2^=0.16 | 0.0051 |
|  | Age | β=-0.014 [-0.036,0.0081] | 0.21 |  |  |
|  | AMH | β=-0.037 [-0.083,0.0079] | 0.10 |  |  |
|  | BMI | β=-0.43 [-0.66,-0.20] | 0.72 |  |  |
|  | Male Factor | β=0.013 [-0.0021,0.028] | 0.94 |  |  |
|  | Change in Total Gonadotropin Dosage Between Cycles | β=-5.29x10^-6^ [-9.27x10^-5^,8.21x10^-5^] | 0.90 |  |  |
| Multivariate with Race/  Ethnicity,  Linear | TVOD | β=-0.50 [-0.77,-0.22] | <0.001 | F=2.83  R^2^=0.35  Adjusted R^2^=0.23 | 0.0096 |
|  | Age | β=-0.026 [-0.052,0.00049] | 0.054 |  |  |
|  | AMH | β=-0.035 [-0.089,0.019] | 0.19 |  |  |
|  | BMI | β=0.017 [-0.00049,0.035] | 0.056 |  |  |
|  | Male Factor | β=0.082 [-0.16,0.32] | 0.49 |  |  |
|  | Change in Total Gonadotropin Dosage Between Cycles | β=-1.99x10^-5^ [-0.00013,8.81x10^-5^] | 0.71 |  |  |
|  | Asian Race/Ethnicity | β=0.057 [-0.31,0.42] | 0.75 |  |  |
|  | Black or African American Race/Ethnicity | β=-0.12 [-0.66,-0.43] | 0.67 |  |  |
|  | Hispanic/Latino  Race/Ethnicity | β=0.057 [-0.30,0.42] | 0.75 |  |  |
